# Guided Growth in Leg Length Discrepancy in Beckwith-Wiedemann Syndrome: A Consecutive Case Series

**DOI:** 10.3390/children8121152

**Published:** 2021-12-07

**Authors:** Maurizio De Pellegrin, Lorenzo Brogioni, Guy Laskow, Graziano Barera, Roberta Pajno, Sara Osimani, Silvia Russo, Lorenzo Marcucci

**Affiliations:** 1 Pediatric Orthopedic and Traumatology Unit, San Raffaele Hospital, 20132 Milan, Italy; brogio@live.it; 2 Orthopedic Residency Program, Vita-Salute San Raffaele University, 20132 Milan, Italy; laskov.guy@hsr.it; 3 Department of Pediatrics, San Raffaele Hospital, 20132 Milan, Italy; barera.graziano@hsr.it (G.B.); pajno.roberta@hsr.it (R.P.); osimani.sara@hsr.it (S.O.); 4 Laboratory of Medical Cytogenetics and Human Molecular Genetics, Research Center, IRCCS Istituto Auxologico Italiano, 20145 Milan, Italy; s.russo@auxologico.it; 5 Orthopedic Residency Program, University of Verona, 37134 Verona, Italy; lore.marcucci93@gmail.com

**Keywords:** BWS, leg length discrepancy, LLD, lateralized overgrowth, temporary epiphysiodesis

## Abstract

Beckwith-Wiedemann Syndrome (BWS) is a rare genetic disorder characterized by overgrowth, macroglossia, abdominal wall defects, neonatal hypoglycemia, predisposition to embryonal tumor, lateralized overgrowth, and leg length discrepancy (LLD), which can affect normal posture and gait. Aim of this study was to evaluate the effects of guided growth (temporary epiphysiodesis technique) as LLD management in BWS patients. Between 2007 and 2021, 22 BWS patients (15 F, 7 M) with a mean age of 7.9 years (2.9–14.4) and a mean LLD at first surgery of 3.65 cm (2–10), underwent temporary proximal tibial (PTE) and distal femur epiphysiodesis (DFE). In 18 patients the first surgical procedure was PTE, in one, DFE, and in 3 cases, PTE and DFE at the same time, respectively. Eleven patients reached equality of leg length after a mean follow-up of 7.7 years (3.7–13.0) and mean age of 13.3 years (12.7–27.5); 10 patients underwent 3 surgical procedures, one 7 procedures. Fifteen patients had no complications. No severe complications, infection, articular stiffness, or neuro-vascular lesions occurred in remaining patients; complications included secondary varus or valgus axial deviation in a total of 6 patients, and two screw breakages in two patients. Guided growth as a minimally invasive procedure seems efficient for LLD treatment with low complication rate in BWS patients.

## 1. Introduction

Beckwith-Wiedemann Syndrome (BWS, OMIM #130650) is a rare (1:10,500) imprinting disorder, mainly in sporadic cases (85% vs. 15% of familial cases) and the clinical diagnosis often appears evident since birth. BWS cardinal features are macroglossia, exomphalos, hyperinsulinism, and embryonal tumors and lateralized overgrowth (LO). Kalish et al. [1] defined LO, or segmental overgrowth, as a significant increase in the length and/or girth of most or all of one side of the body compared to its contralateral side. It replaces “hemihyperplasia” and “hemihypertrophy” because the term “hemi” seems to indicate that the overgrowth should be present at the same half of the body. However, overgrowth can also be present in body parts that differ in body laterality. Brioude et al. in 2018 introduced the concept of BWS spectrum (BWSp) to summarize different BWS phenotypes with or without segmental overgrowth [2].

The molecular etiology of BWS spectrum consists in the (epi)genetic deregulation at two loci (IC1 and IC2) within 11p15.5 region. Over 50% of BWS patients show loss of methylation (LoM) at the maternal IC2, 20% paternal uniparental disomy of chromosome 11 (UPD(11)pat), while maternal IC1 gain of methylation (GoM) and CDKN1C gene mutations account for 5% of patients, respectively [2]. A genotype-phenotype correlation was reported, with upd(11)pat and IC1 conferring the higher tumor risk respectively 13.85 and 22.8%. A genotype-phenotype correlation was reported, with upd(11)pat appearing as the molecular defect more associated with LO [3].

**Figure 1:**
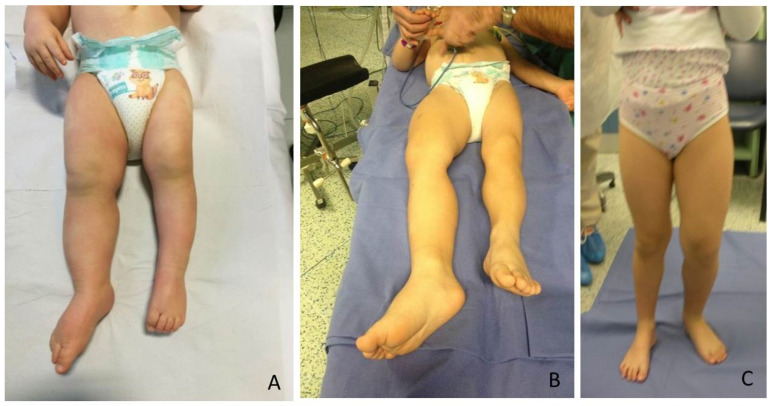
BWS (Beckwith-Wiedemann syndrome) patients at different ages with LO (lateralized overgrowth) and LLD (leg length discrepancy) involving both femur and tibia: (**A**) nine-month-old neonate with LO and mild (<2 cm) LLD right; (**B**) three years old male child with critical (>5 cm) LLD right; (**C**) eight-year-old female child with severe (2–5 cm) LLD left.

The most relevant orthopedic feature is LO, which can cause leg length discrepancy (LLD) involving both the femur and tibia (Figure 1). In literature, only 43–65% of patients are reported to have LLD [4].

LLD can be the cause of abnormal posture and gait, causing knee, hip and low back problems and influence common activities when severe [5]; LO may not be always present though. Mussa et al. [4] reported that only one third of BWS patients develop LO. In the general population, the prevalence of LLD is higher, approximately 70%, but only 1/1000 have more than 2 cm of difference [5]. IC1 and upd(11)pat mutations were linked to higher likelihood of developing LLD as well as those patients showing LLD already at birth. Both usually have a worse evolution over time than other BWSp and Isolated Overgrowth [6].

The management of LLD depends on its severity: shoe-lift is an option for minor discrepancies (less than 1.5 cm); in more severe LLD, the two main surgical options are temporary or definitive epiphysiodesis or leg lengthening of the femur and tibia [6]. Among temporary epiphysiodesis techniques, Stevens [7] introduced the concept of guided growth, which means a selective temporary and reversible blockage of the growth plate of the affected bones.

Furthermore, postnatal growth in BWS children is generally in the upper percentiles of the normal range and differences in growth trajectories should be considered in BWS orthopedic treatment [6].

The aim of this study was to evaluate the effects of guided growth for LLD management in BWS patients. This case series gathered a large number of patients affected by a rare disease and treated by the same orthopedic surgeon. Guided growth as a minimally invasive procedure [7] seems efficient for LLD treatment with low complication rate in BWS patients and poor effects on height growth.

## 2. Materials and Methods

Including criteria: BWS genetic or BWS clinical diagnosis associated with LLD, availability of data about LLD, and height before the first surgical procedure and of the same data at the most recent follow-up or at the end of it. Excluding criteria were genetic diagnosis that excludes BWS pattern, such as isolated hemihypertrophy, and the impossibility to collect correct measurement before and after surgery.

Between 2007 and 2021, 22 patients, 15 F and 7 M (IC1 (*n* = 3), IC2 (*n* = 7), upd(11)pat (*n* = 6), clinical diagnosis (negative or not determined yet genetic test) (*n* = 4)), who underwent temporary epiphysiodesis (TE) according to guided growth technique, were consecutively included in this study (Table 1). In 14 cases, the right lower limb was involved while in 8 cases the left legs were involved.

**Figure 2:**
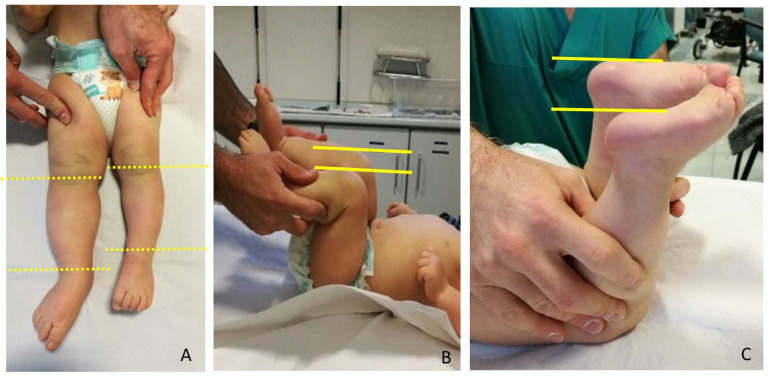
Clinical LLD (leg length discrepancy) evaluation in neonates: (**A**) Femur and tibia were measured singularly, from anterior superior iliac spine (not signed) to medial knee joint rime (dotted line) and from latter to medial malleolus (dotted line); (**B**) in supine position with 90° flexed hips and knees, femur length discrepancy can also be evaluated; (**C**) in prone position with extended hips and 90° flexed knees, tibia length discrepancy can also be evaluated.

**Figure 3:**
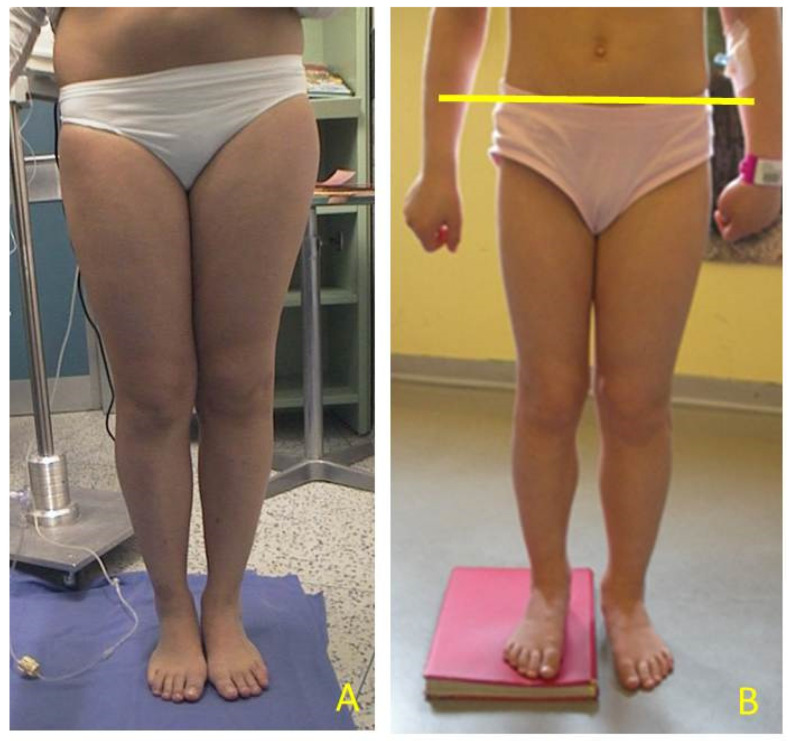
Eight-year-old girl with BWS (Beckwith-Wiedemann syndrome) and LO (lateralized overgrowth) left and mild LLD (leg length discrepancy): (**A**) LLD causes pelvic obliquity and abnormal back posture; (**B**) correction simulating shoe lifts to equal LLD and pelvic position. Height was also measured with and without a shoe-lift under shorter leg.

LLD evaluation was clinically screened by a direct method (tape-measure method). Each lower extremity was measured from the anterior superior iliac spine to the medial malleolus. Femur and Tibia were measured singularly too, from anterior superior iliac spine to medial knee joint rime and from the latter to medial malleolus, respectively (Figure 2). Height was also measured with and without a shoe-lift under the shorter leg. In this study we consider correct only those measures carried out without shoe-lift under the normal shorter leg (Figure 3).

Guided growth consists in performing epiphysiodesis using two plates and four screws for each growth plate: implanting one plate and two screws for the medial and one plate and two screws for the lateral aspects of the growth plate allows it to stop its growth. The implants used in these cases were titanium plates (8-plate^R^ and quad-plate^R^; Eight Plate Guided Growth System+, Orthofix Medical Inc.©, Lewisvill, TX (USA)) and noncannulated titanium screws. This implant constitutes a flexible tension band that guides growth instead of exerting compression forces like previous techniques [8]. The technique according to Stevens was adopted: 8-shaped plates of 12 mm or 16 mm dimension, chosen according to patient’s age and bone dimensions were centered over the physis using a K wire under fluoroscopic control and fixed to the bone with a metaphyseal and epiphyseal screw without damaging periosteum and the growth plate. If the screws are placed parallel, they tend to diverge over the first few months, indicating a lag effect. It is preferable to place them moderately diverging for length inhibition to reduce lag time [9]. The concept of serial guided growth may be applied for both the ipsilateral angular deformities and to decelerate the growth in the contralateral, longer limb. Once the healthy limb reaches the length of the affected one, the plate used for the temporary epiphysiodesis has to be removed to avoid an overcorrection on the contralateral side.

**Figure 4:**
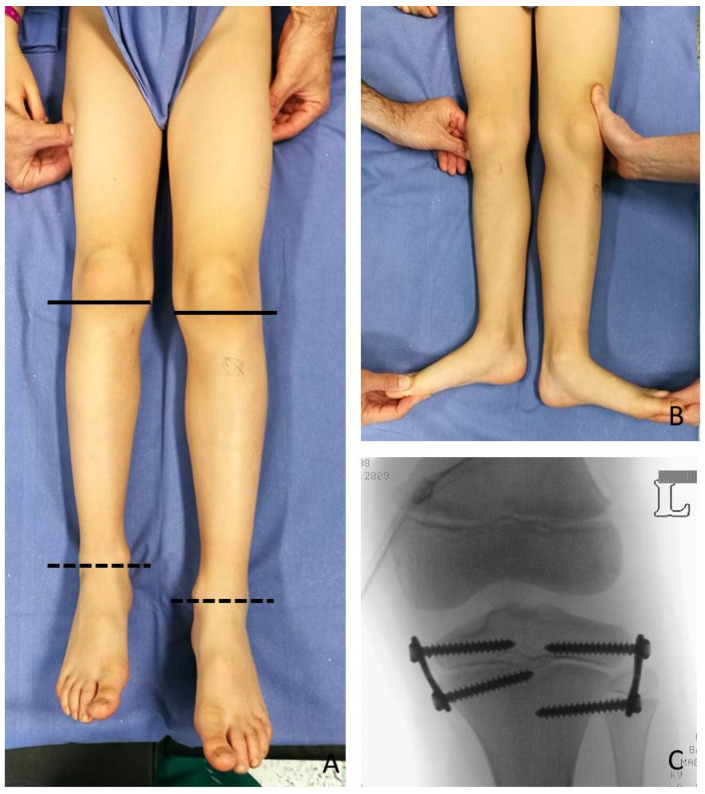
BWS (Beckwith-Wiedemann Syndrome) male patient with LLD (leg length discrepancy) left at age of 6 years: (**A**) for clinical evaluation, medial knee joint rime (whole lines) and medial malleolus (dotted line) of both legs are signed; only mild length discrepancy of femurs but severe discrepancy of tibias are present; (**B**) in external rotation of both lower extremities LLD is clearly visible; (**C**) postoperative X-ray in antero-posterior projection showing performed PTE (temporary proximal tibia epiphysiodesis) with implanting of one plate and two screws for medial and one plate and two screw for lateral aspects of growth plate respectively to stop growing of proximal tibia (8-plate^R^, Eight Plate Guided Growth System+, Orthofix Medical Inc.© Lewisville, TX (USA)).

**Figure 5:**
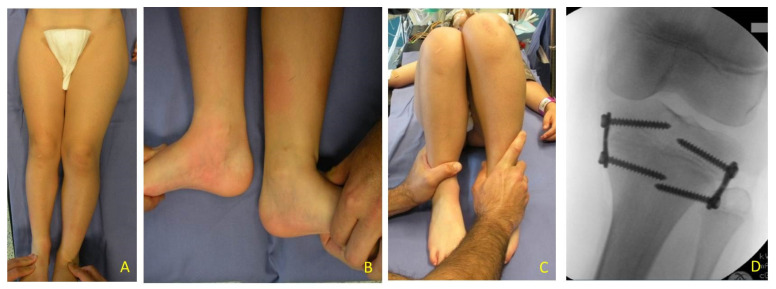
Same patient as shown in Figure 3, one year later: (**A**) LLD (leg length discrepancy) increased; (**B**) a severe LLD of 4 cm is present; (**C**) tibia shows major discrepancy; (**D**) postoperative X-ray showing performed PTE (temporary proximal tibia epiphysiodesis).

**Figure 6:**
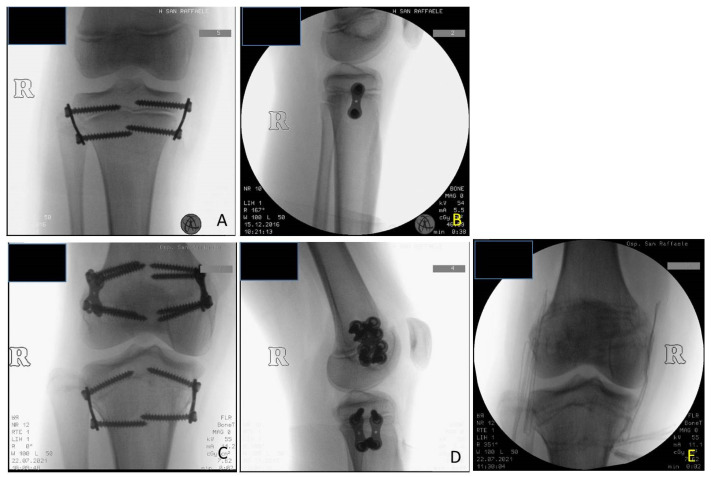
BWS (Beckwith-Wiedemann syndrome) female patient with LLD (leg length discrepancy) right: (**A**) at age of 9.8 years PTE (temporary proximal tibia epiphysiodesis) was performed using 8-plates; X-ray in anteroposterior projection (**B**) X-ray in lateral projection; (**C**) at age of 10.7 years DFE (distal femur epiphysiodesis) was performed using quad-plates, X-ray in antero-posterior projection; (**D**) X-ray in lateral projection; (**E**) at age of 14.3 years removal of hardware by absent LLD, X-ray in antero-posterior projection showing closed physis. (8-plate^R^ and quad-plate^R^; Eight Plate Guided Growth System+, Orthofix Medical Inc.© Lewisvill, TX (USA)).

**Figure 7:**
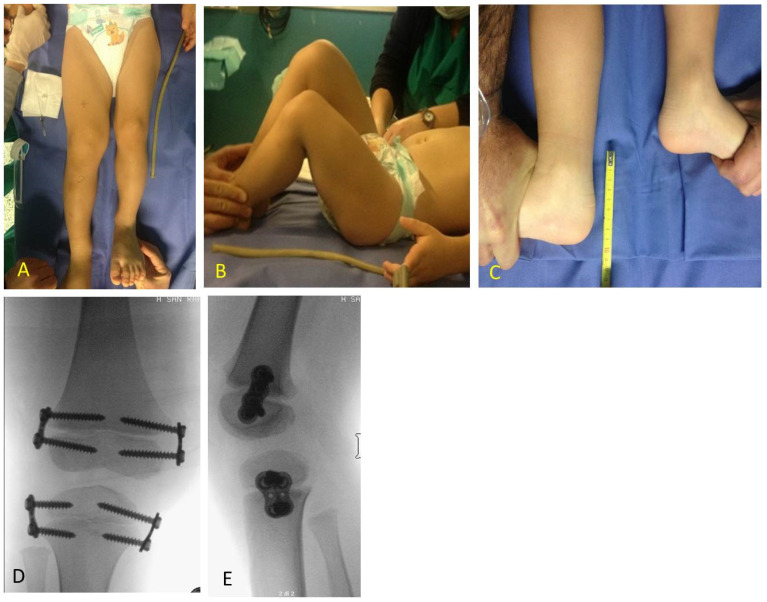
BWS male child with LO (lateralized overgrowth) right and severe LLD (leg length discrepancy): (**A**) clinical aspect showing both femur and tibia length discrepancy; (**B**) femur and tibia length discrepancy is clearly visible from lateral with flexed hips and knees; (**C**) LLD of 10 cm is present; (**D**,**E**) PTE (temporary proximal tibia epiphysiodesis) and DFE (distal femur epiphysiodesis) were performed in one step at same time at age of 2.11 years, X-ray in antero-posterior projection showing position of guided growth system with 8-plates straddles distal femoral physis and proximal tibial physis.

**Figure 8:**
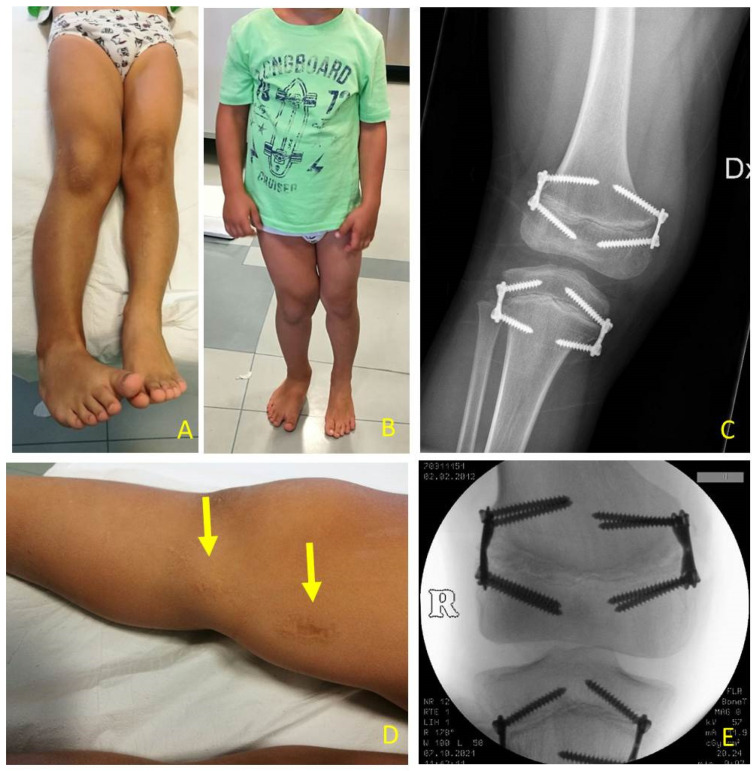
Same patient as shown in Figure 7, at age 6.7 years: (**A**) LLD (leg length discrepancy) decreased but valgus deformity is developing; (**B**) clinical aspect in standing position; (**C**) X-ray of right knee shows valgus deformity and divergent aspects of screws; (**D**) small skin scars after first surgical procedures (yellow arrows); (**E**) X-ray on knee in antero-posterior projection shows on femur substitution of eight-plate with stronger Quad-plates to avoid increasing valgus deformity (8-plateR and quad-plateR; Eight Plate Guided Growth System+, Orthofix Medical Inc.© Lewisvill, TX (USA)).

**Figure 9:**
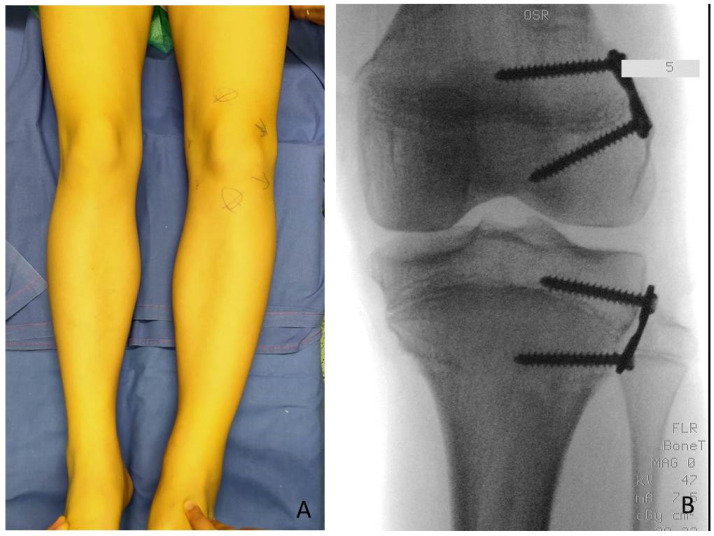
Fourteen years old girl with BWS (Beckwith-Wiedemann syndrome) and increasing varus deformity after TE (temporary epiphyseodesis) of femur and tibia: (**A**) clinical aspect showing varus deformity of left knee; (**B**) PTHE (proximal tibia hemiepiphysiodesis) and DFHE (distal femur hemiepiphysiodesis) were performed on same time to correct deformity during remaining growth time.

At the time of the first surgical procedure, mean age was 7.94 years (range 2.91–14.41) and mean LLD was 3.65 cm (range 2–10). TE was performed either on the growth plate of the proximal tibia (PTE) and on the growth plate of the distal femur (DFE) (Figure 4, Figure 5 and Figure 6). Due to the different distribution of LLD with the tibia more and early involved, usually the first step was to treat proximal tibial growth plate, then followed by the distal femur in a second surgical procedure (Figure 6). If LLD was very severe at first evaluation or if growth potential was very high during clinical follow-up, PTE and DFE were performed in one step at the same time (Figure 7). If, during guided growth treatment lower limb axial deviation (varus-valgus deformity) occurs, further surgical procedures were necessary, such as removal and changing of the implants (Figure 8), proximal tibia hemiepiphysiodesis (PTHE) or distal femur hemiepiphysiodesis (DFHE) (Figure 9).

Among the 22 patients, 27 tibial 8-plate implants, 16 femoral 8-plate implants, 7 femoral quad-plate implants, and 1 tibial quad-plate implant according to TE technique were performed. When considering the further surgical procedures necessary for correction of LLD and axial deviation during treatment and removal of implants at the end of treatment, a total of 90 surgical procedures in 61 surgical sessions were performed. The number of surgical procedures performed for each patient varied according to LLD severity and deformities which occur during the growth process after the first TE. It varies from a minimum of 3 surgical procedures: PTE, DFE and implant removal, to a variable amount if deviations in mechanical axis occur (PTHE and DFHE). TE can be modified in hemiepiphysiodesis by removing the medial or lateral plate in varus or valgus deformity respectively (Figure 9). The maximum number of surgical procedures we registered for a single patient was 7.

To analyze the impact of TE in BWSp patients’, we compare using Wilcoxon test, a nonparametric statistical test, growth height percentiles, measured height before performing surgery and at the most recent follow-up evaluation, of 20 patients. Two patients were excluded because of incomplete data. To reduce geographical factors which may influence children’s growth, WHO growth charts were used. The follow-up length was calculated from the day of the first surgical procedure to the last day the patient was evaluated till the end of this study.

## 3. Results

The mean follow-up of all the patients in the study was 5 years and 7 months (range 6 months–13 years); it rose to 7 years and 3 months (range 3–13 years) when considering only those patients who already reached the end of the intervention.

Among 22 patients treated for LLD the following procedures were performed as the first surgical step: 18 PTE, 1 DFE and 3 PTE + DFE at the mean age of 7 years and 11 months (range 2 years and 11 month–14 years and 5 months). As second surgery, DFE was performed in 11 patients at a mean age of 10 years and 2 months (range 8 years, 5 months–12 years, 4 months) including all patients who underwent DFE firstly, then underwent PTE. 

In one case of a girl who had 3.5 cm of LLD at the age of 9 years, a DFE was performed as the first procedure and PTE as the second procedure because of the low growth potential. After successful correction all implants were removed in a third procedure after 2.5 years. In one case of a boy with 10 cm of LLD at the age of 2.11 years TE with PTE and DFE was performed in one step despite the young age (Figure 7).

The implants were definitively removed in 11 patients at the mean age of 13 years and 3 months (range 10 years and 8 months–17 years) and a mean follow-up of 5 years (2 years and 3 months–11 years and 3 months) (Figure 6).

A residual LLD of 1 cm was present in two patients (as overcorrection with a shorter leg on the LO side in one case and with a residual plus on the LO side in another case). No treatment with shoe-lift was necessary.

**Figure 10:**
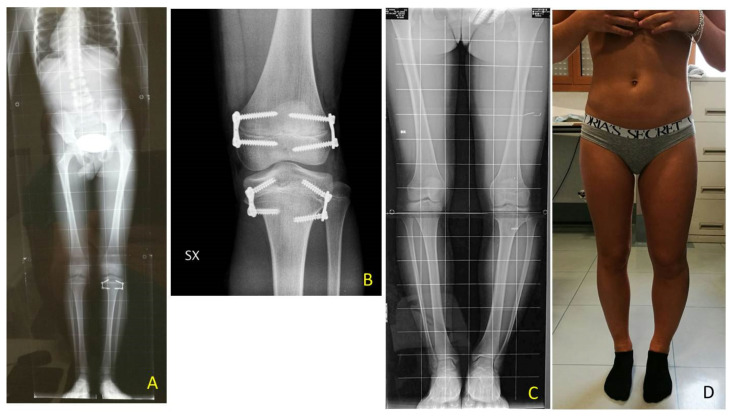
BWS (Beckwith-Wiedemann syndrome) female patient with severe LLD (leg length discrepancy) left. At age of 7 years with a length discrepancy of femur of 1.5 cm and of 3.5 cm of tibia a PTE (temporary proximal tibia epiphysiodesis) was performed: (**A**) at age of 9.4 years femur discrepancy of 3.0 cm was present leading to a severe pelvis obliquity without shoe lift correction as shown in X-ray od spine and lower extremities; (**B**) postoperative X-ray in antero-posterior projection showing performed DFE (temporary distal femur epiphysiodesis); (**C**) at age of 12.7 years, after reaching leg length equality, removal of implants was performed. X-ray showing screw breakage on lateral aspect of tibia; (**D**) clinical aspect at follow-up with mild varus knee deformity bilaterally and nonpelvic obliquity.

Temporary epiphysiodesis’s main complications were: 14 implant migrations with secondary varus-valgus axial deviation in a total of 6 patients. Hemi-epiphysiodesis was necessary to correct axial deviation that occurred during growth. To have a greater effectiveness in growth plate block, new bigger “quad” plates were used (8) in 6 cases. In only two cases screw breakage occurred during surgical removal (Figure 10).

In 6 patients, a revision of the plate’s configuration was necessary. Due to plate migration the eight plates were re-implanted. When axial deviation occurred, hemiepiphysiodesis was performed. In the first follow-up, after the first surgical procedure, 4 varus axial deviations and 1 plate migration occurred. After the second procedure, 3 valgus and 1 varus axial deviations, 1 plate migration, and 2 screw breakages occurred.

Neither severe complications, such as infection, articular rigidity of neuro-vascular lesions, nor minor complications were found. No growth arrest or impossibility to restart daily activities occurred. 

All the tibial and femoral plates were removed in 11 patients after LLD final correction: 10 patients underwent 3 surgical procedures, one a total of 7 procedures; 8 patients had no complications; 6 patients had axial deviation and 2 patients screw breakage.

The height percentiles of a total of 20 patients were analyzed: the mean height at the evaluation before the first surgical approach and at the most recent FU evaluation was settled between the 75th (0.67 SDS) and 90th (1.28 SDS) percentiles, with a mean SDS of 1.25 and 0.86, respectively. No significant statistical difference was found (*p*-value 0.30302) using the Wilcoxon test between height percentiles before and after surgery.

Mean height at the end of treatment (all plates removed) was 166.15 cm. Among the eleven patients who reached the end of treatment only one was a boy. The 11 girls’ mean initial height was 138.11 cm (SDS 1.34) and at the plate removal was 167.51 cm (SDS 1.02) at a mean age of 13 years and 4 months.

Data of the patients with results and complications after surgical treatment using guided growth are reported in Table 2.

## 4. Discussion

BWS is a rare genetic disorder with a variable patient genetic spectrum and a difficulty to define a unique and homogeneous clinical phenotype. A recent international consensus [2] introduced the term “lateralized overgrowth” (LO) to summarize different BWS phenotypes when segmental overgrowth is present. LO can lead to LLD, which represents the most relevant orthopedic concern. Both tibia and femur overgrowth are present and vary from case to case. LLD which can affect normal posture and gait, hip, and back alignment, with possible difficulties in walking, running, and practicing sport activities according to its severity. In our study, genetic diagnosis distribution IC2 (*n* = 7) and upd(11)pat (*n* = 6) with the higher risks of tumor [6] represented the majority of the cases. In our case series, no one developed tumors during guided growth follow-up and LLD was dramatically reduced.

Even though a treatment flowchart was made by the BWS Italian scientific committee [10], in literature there is no consensus on the best management of LLD in BWS patients. As no BWSp specific growth pattern was ever described, treatment strategies are usually chosen according to each orthopedic surgeon’s experience [6]. According to the Italian Committee’s operative flowchart, LLD can be categorized into four groups based on differences in length: minor, <1 cm; mild, 1–2 cm; severe, 2–5 cm; critical ≥5 cm [10]. In case of mild LLD, treatment mostly consists of shoe-lifts to equal LLD. When LLD is >2 cm surgery may be considered and can include epiphysiodesis for growing children and, less frequently, bone lengthening for adolescents. The decision making surrounding the indications and timing of epiphysiodesis is challenging because of the unpredictable final discrepancy, especially in young children [11].

Despite the unknown velocity of growth of the affected femur and tibia in BWS those data were also considered for the timing of surgical correction using guided growth. Timing is crucial for epiphysiodesis to avoid over or under correction, especially in definitive nonreversible techniques. When calculating definitive timing modern algorithms represent important advances, a margin of error is still present [4] particularly if blockage of growth should be performed at an early age due to the severe LLD (Figure 7).

In BWS patients considering the shorter or the longer limb, management of LLD distinguishes between lengthening (using external fixators according to Ilizarov technique) of the “normal” one or shortening with surgical stop the pathological longer limb growth (temporary or definitive epiphysiodesis).

The major risk of definitive epiphysiodesis is to perform it too early resulting in overcorrection and so a shorter limb than the contralateral normal one. Temporary epiphysiodesis should be preferred due to its minor risk as growth plates are not damaged. Performing surgery too late could expose to the risk of not having enough residual growth to equal contralateral limb length, if it is performed too early once the devices are removed LLD could relapse.

Limb lengthening (LL) is also considered by orthopedic surgeons. LL is usually associated with a higher rate of superficial and deep tissue infections, neuro-vascular damage, articular rigidity, pain, and slower return to daily activities as recovery is longer and the patient is not independent. Complications are common, sometimes serious, and often require unanticipated secondary procedures [7].

When performing LL functional aspects must be considered: articular range of movement, pain, lameness, and everyday activity restrictions [12]. Nerve paralysis may be caused by soft tissue stretching, compartmental syndrome or surgical procedure itself [13,14]. Galardi et al. [15] showed that all 5 patients studied had nerve damage due to LL.

**Figure 11:**
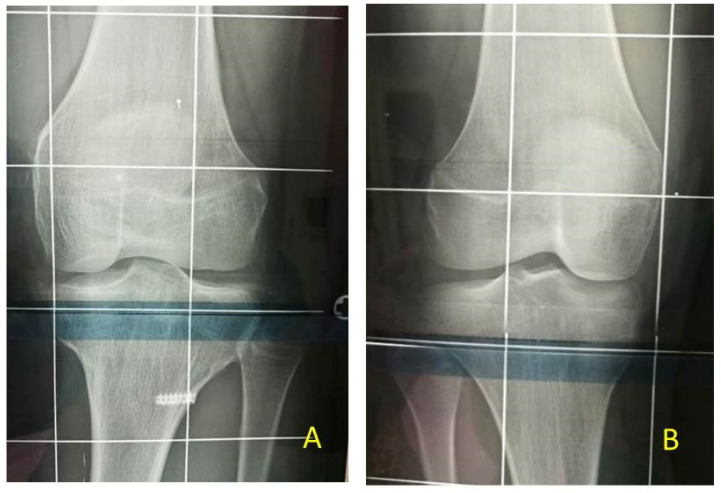
Same patient shown in Figure 10: (**A**) X-ray of left knee at follow-up after PTE (temporary proximal tibia epiphysiodesis) and DFE (temporary distal femur epiphysiodesis). No change in morphology of tibial plateau during growth; (**B**) X-ray of right knee for comparison.

Concerning the risks of TE technique, some authors introduced the concept that the 8-plate epiphysiodesis may cause a “volcano” type deformity, a change in the morphology of the tibial plateau. This deformity occurs due to a change in metaphyseal-epiphyseal angle as evidenced by splaying of the screw during the process as a consequence of continued growth in the central part of the physis. This bony deformity may potentially cause joint incongruity and joint laxity [16]. In our patients at the end of treatment we did not see any morphological changes of the tibial plateau (Figure 6 and Figure 11).

Choosing to treat fibula is an issue in epiphysiodesis planning. Many surgeons prefer to avoid performing proximal fibular epiphysiodesis in LLD because of the risk of peroneal nerve injury [8]. Others suggest performing it if desired tibial correction is >2 cm [8,17] or if fibular overgrowth is estimated to exceed 2 cm [6,18] to prevent relative overgrowth of the fibula. Boyle et al. [19] conducted a study on 234 patients to understand if proximal fibular epiphysiodesis matters. 179 patients had undergone concomitant fibular epiphysiodesis and 55 had not. According to their study, they did not find significant fibular overgrowth in patients undergoing proximal tibial epiphysiodesis without concomitant PFE; performing a PFE did not ensure the prevention of fibular overgrowth. One of the different hypotheses dealing with fibula overgrowth is that in patients without PFE there could be a secondary passive deceleration of fibular growth [19].

In patients of our study, we never perform proximal fibular epiphysiodesis. The 11 patients at final FU did not have significant overgrowth of the proximal fibula at X-ray evaluation (Figure 6, Figure 9 and Figure 10). Our hypothesis is that the strong tibio-fibular proximal joint ligaments avoid the overgrowth of fibula during PTE.

Based on our experience, to correct LLD at least three surgical procedures are needed: the first at 7–8-years-old at the tibia, the second at 10–11-years-old to the femur, and the third one to remove all the plates once LLD was corrected. However, drift in mechanical axis may occur during growth, and so more surgical procedures could be needed. Our results show that in 22 patients, only 6 patients had varus or valgus deviation. More surgical procedures (maximum 7 in a single patient) were needed to modify growing direction.

In literature there is a lack of data about surgical follow-up in BWSp patients treated with TE. We analyzed a total of 22 patients; 11 patients ended the follow-up for definitive LLD resolution. 

TE can be modified in hemi-epiphysiodesis by removing medial or lateral plate in varus or valgus deformity respectively. The maximum number of surgical procedures we registered for a single patient is 7. This case is representative for the adaptability of guided growth which allows to correct LLD and axial deviations.

Guided growth process needs close surgical follow-up to avoid overcorrection or axial drifts. The frequency of clinical evaluation depends on the patient’s age and the supposed growth velocity: greater velocity means closer evaluation. Mean FU duration in our study was 5 years and 7 months considering all the patients. However, it rises to 7.74 years considering only patients who have reached the end of treatment. Follow-up time varies because in this study there are children at different correction timings.

The second orthopedic concern is BWS patients’ height. In literature it is reported that postnatal growth in BWSp patients is usually in the higher percentiles of growth chart and slows down in late childhood [2]. There are no specific growth charts or standardized measurement techniques. Bone age is higher compared to the chronological age in rare cases (about 3%) [20,21]. As highlighted in a study by Brioude et al. [22] there are several articles in the literature that report data from small cohorts of patients with discordant results: some do not record an average height greater than 2 SDS; others, on the other hand, report an increase in the average height of BWSp patients, indicating that the final height in adulthood tends to be greater than the target height calculated on parental stature, with an average difference of 1.7 ± 1.1 SDS and that about half of patients have a height with SDS > 2. Our unpublished study reported that predicted final adult height in 48 BWS patients was higher than parental target height, with a mean target of 174.58 ± 10.15 SDS and 169.55 ± 8.01 SDS respectively, and there was a statistical significative difference proved by Wilcoxon test (*p*-value < 0.05).

Parents usually object to the idea of stunting their child’s growth [7], so we analyzed children height percentiles before and after surgery to find if this procedure may negatively influence children’s growth. Height and the corresponding percentile right before surgery and at the very last follow-up evaluation available were compared with a non-parametric statistical test (Wilcoxon test). No statistical difference was found (*p*-value = 0.30302) between the two groups of percentiles. We could assume that TE does not affect children’s growth, which continues to be in the upper percentiles.

Among our 22 patients included in this study, few complications occurred. The most frequent is axial deviation due to 8-plate failure, a condition that occurs in only 6 patients. In some cases, a quad plate, instead of the classical 8-plate, was used to achieve a stronger blockage of the growth plate. In two cases, screw breakage occurred during removal procedures and the fragment was left inside the bone as it does not affect the growth plate. No case of deep or superficial infections, treatment failure, or disposal intolerance were registered. LL would have major complications, as said before. Despite the complications that may occur, all 12 patients that ended the FU reached the same length of the lower limb.

A true cause-effect result of intervention could not be evaluated due to study design and to the low incidence of this syndrome and the difficulty in gathering all the orthopedics cases in one referral center. Clinical follow-up may be less accurate when measurements are not all done by the same doctor. In our study, this is partially true because the orthopedic follow-up was made by the same surgeon and therefore axial deviation diagnosis was standardized. As a retrospective study we encounter some difficulties to gather some data of the clinical measurements during the follow-up. Our results should be compared to another population with LO to describe more objectively BWSp complication frequency and growth pattern. A longer FU would help in having a larger database and a more accurate statistical analysis, but as BWS is a rare syndrome this would take several years.

The strengths of this case series are that, to the best of our knowledge, it is the most numerously described gathering of clinical and genetic phenotypes and surgical outcomes with a long follow-up in the literature. Fifty percent of our patients ended follow-up reaching skeletal maturity; all of them showed leg length equality. These surgical procedures are standardized and there were no severe complications. Surgical procedures were performed by the same surgeon so the outcomes can be analyzed objectively.

Height, tibia, and femur growth charts and standardized height measurement techniques are needed to develop the best LLD management.

## 5. Conclusions

Management of LLD in BWS patients is challenging, and no guidelines are present in literature. In more severe LLD, the two main surgical options are shortening by definitive or temporary epiphysiodesis of the femur and tibia growth plates or leg lengthening, according to Ilizarov technique. Leg lengthening, which considers treatment of the “normal” leg, shows a high rate of complications and often requires secondary procedures. Shortening considers treatment of the pathological longer limb and can be performed as a definitive or temporary procedure. Timing is crucial for epiphyseodesis to avoid over- or under-correction, especially in definitive, nonreversible techniques. Temporary epiphysiodesis following the concept of guided growth, which means a selective temporary and reversible blockage of the growth plate of the affected bones, is a minimally invasive procedure with low complication rate, so it seems to be the best choice for the treatment of LLD also in relation to patient’s age, predicted final height, and LLD severity.

## Figures and Tables

**Table 1:** Preoperative data of patients affected by BWS and LLD. F: female, M: male; IC2: maternal IC2 loss of methylation, IC1: maternal IC1 gain of methylation, upd(11)pat: uniparental disomy of chromosome 11, R: right, L: left; y: years, m: months; n.d.: not determined; n.a. not available.

Patient	Sex	Genetics	Side	Age at 1st Surgery (Months)	Heigth at 1st Surgery (cm)	Lld at 1st Surgery (cm)
1	F	IC2	R	10y 3m	154	3.5
2	F	IC1	R	8y 6m	127.8	3
3	F	n.d.	R	9y 1m	141.3	3.5
4	F	IC2	R	6y 6m	126.8	3
5	M	IC2	R	9y 3m	137.2	2.5
6	F	n.d.	L	14y 5m	n.a.	2
7	F	n.d.	L	6y 7m	122.2	4
8	M	IC2	L	8y 1m	n.a.	4
9	M	upd(11)pat	R	2y 11m	107	10
10	M	Negative	R	5y 10m	117.8	3
11	F	upd(11)pat	R	7y 10m	130.7	4
12	F	IC2	L	8y 11m	149.6	2.5
13	F	IC2	R	9y 8m	140.5	3.5
14	M	upd(11)pat	R	9y 4m	140.5	3
15	F	upd(11)pat	L	7y	/	5
16	F	upd(11)pat	R	5y 8m	111.5	3
17	M	Negative	R	4y 11m	117	6
18	F	upd(11)pat	R	9y 6m	132	3
19	F	n.d.	L	9y 6m	145	2.5
20	F	IC1	L	10y	158	3.5
21	F	IC2	L	8y 2m	147	3
22	M	IC1	R	8y 9m	137	3

**Table 2:** Data of BWS patients with LLD who underwent guided growth. TE: temporary epiphysiodesis; PTE: proximal tibial epiphysiodesis; DFE: distal femur epiphysiodesis; DFHE: distal femur hemiepiphysiodesis; PTHE: proximal tibial hemiepiphysiodesis; FU: follow-up; y: years, m: months. Revision: plate changing to correct axial deviations; n.a. not applicable because surgery has not been performed yet. Residual LLD values: negative: overcorrection; positive: undercorrection.

Patient	1st TE	2nd TE	Age at 2nd TE	Definitive Disposal Removal	Age at Definitive Disposal Removal	Complications	Total N° of Surgeries	FU	Age at Last FU	Heigth at Last FU (cm)	FU End (Yes/No)	Residual LLD (cm)
1	PTE	DFE	12y 4m	yes	13y 4m		3	4y 7m	14y 11m	174	yes	0
2	PTE	DFE	11y 6m	yes	13y 6m	Valgus	3	5y	13y 7m	156	yes	0
3	DFE	PTE	10y 5m	yes	11y 10m		3	6y 2m	15y 5m	171	yes	0
4	PTE	DFE PTHE	8y 5m	no	n.a.	Valgus Varus	5	5y 3m	11y 10m	144.6	no	
5	PTE	/	n.a	no	n.a.		1	2y 11m	12y 2m	155	no	
6	PTE	DFE		yes	16y 3m		3	13y 1m	27y 7m	175	yes	+1
7	PTE	DFE	8y 7m	yes	10y 8m		3	9y 6m	16y 1m	165	yes	0
8	PTE	/	n.a.	no	n.a.		1	4y 4m	12y 6m	157	no	
9	PTE DFE	PTE DFHE	6y 7m	no	n.a.	Valgus Varus	3	6y 3m	9y 5m	136	no	
10	PTE	DFE	9y 4m	yes	12y 6m		3	7y 3m	13y 1m	152.6	yes	0
11	PTE DFE	PTE (revision)	9y 9m	no	n.a.	Screw migration Valgus Varus	5	6y 2m	14y 2m	163	no	
12	PTE	PTHE (quad-plate)	10y 10m	yes	11y 8m	Valgus	3	3y 10m	12y 9m	176	yes	0
13	PTE	DFE	10y 7m	yes	14y 3m		3	4y 7m	14y 4m	168	yes	0
14	PTE	/	n.a.	no	n.a.		1	1y 6m	10y 11m	151	no	
15	PTE	DFE	9y 4m	yes	12y 7m	Screw breakage	3	11y 3m	18y 3m	160	yes	−1
16	PTE	DFE	10y 6m	yes	16y 11m	Valgus Varus Screw breakage	7	11y 3m	17y	159	yes	0
17	PTE	/	n.a.	no	n.a.		1	6m	5y 6m	118.5	no	
18	PTE	/	n.a.	no	n.a.		1	1y 1m	10y 7m	140	no	
19	PTE	DFE (quad-plate)	10y 10m	no	n.a.		2	3y 11m	13y 5m	165	no	
20	PTE	PTE (revision)	11y 1m	yes	12y 9m		3	3y 8m	12y 9m	171	yes	0
21	PTE	DFE	10y 2m	no	n.a.		2	2y 1m	10y 4m	164	no	
22	PTE	/	n.a.	no	n.a.		1	2y 8m	11y 6m	157	no	

## Data Availability

The datasets used and/or analyzed during the current study are available from the corresponding author on reasonable request.
